# Germline hemizygous deletion of *CDKN2A–CDKN2B* locus in a patient presenting with Li–Fraumeni syndrome

**DOI:** 10.1038/npjgenmed.2016.15

**Published:** 2016-06-01

**Authors:** Sock Hoai Chan, Weng Khong Lim, Scott T Michalski, Jing Quan Lim, Nur Diana Binte Ishak, Marie Met-Domestici, Cedric Ng Chuan Young, Karen Vikstrom, Edward D Esplin, Jennifer Fulbright, Mei Kim Ang, Joseph Wee, Kesavan Sittampalam, Mohamad Farid, Stephen E Lincoln, Koji Itahana, Syafiq Abdullah, Bin Tean Teh, Joanne Ngeow

**Affiliations:** 1 Cancer Genetics Service, Division of Medical Oncology, National Cancer Centre Singapore, Singapore, Singapore; 2 Laboratory of Cancer Epigenome, Division of Medical Sciences, National Cancer Centre Singapore, Singapore, Singapore; 3 Cancer and Stem Cell Biology Program, Duke-NUS Graduate Medical School Singapore, Singapore, Singapore; 4 Invitae, San Francisco, CA, USA; 5 Division of Radiation Oncology, National Cancer Centre Singapore, Singapore, Singapore; 6 Department of Pathology, Singapore General Hospital, Singapore, Singapore; 7 RIPAS Hospital, Bandar Seri Begawan, Brunei, Darussalam; 8 Cancer Science Institute of Singapore, National University Singapore, Singapore, Singapore; 9 Institute of Molecular and Cellular Biology, A*STAR, Singapore, Singapore; 10 Oncology Academic Clinical Program, Duke-NUS Graduate Medical School, Singapore, Singapore

## Abstract

Li–Fraumeni syndrome (LFS) is a rare cancer predisposition syndrome usually associated with *TP53* germline alterations. Its genetic basis in *TP53* wild-type pedigrees is less understood. Using whole-genome sequencing, we identified a germline hemizygous deletion ablating *CDKN2A–CDKN2B* in a *TP53* wild-type patient presenting with high-grade sarcoma, laryngeal squamous cell carcinoma and a family history suggestive of LFS. Patient-derived cells demonstrated reduced basal gene and protein expression of the *CDKN2A*-encoded tumour suppressors p14^ARF^ and p16^INK4A^ with concomitant decrease in p21 and faster cell proliferation, implying potential deregulation of p53-mediated cell cycle control. Review of 13 additional patients with pathogenic *CDKN2A* variants suggested associations of germline *CDKN2A* mutations with an expanded spectrum of non-melanoma familial cancers. To our knowledge, this is the first report of a germline gross deletion of the *CDKN2A–CDKN2B* locus in an LFS family. These findings highlight the potential contribution of germline *CDKN2A* deletions to cancer predisposition and the importance of interrogating the full extent of *CDKN2A* locus in clinical testing gene panels.

## Introduction

Li–Fraumeni-like (LFL) syndrome is a variant of the Li–Fraumeni syndrome (LFS), a rare autosomal dominant cancer predisposition syndrome characterised by early onset of cancer and a broad tumour spectrum.^1^ Patients with LFS/LFL typically present with characteristic LFS-associated tumours (soft-tissue sarcoma, osteosarcoma, breast cancer, brain tumour, leukaemia and adrenocortical carcinoma). The majority of these patients have germline mutations in *TP53*, a known susceptibility gene associated with LFS/LFL.^2^ Studies have found that up to 20% of LFS^1,2^ and about 60% of LFL^3^ families are wild-type for *TP53.* Germline alterations of other genes such as *CHEK2* and *CDKN2A* in LFS/LFL families without *TP53* mutations have been reported, but their role as susceptibility genes as yet remains controversial.^1,2^

Here we describe a specific patient presenting with both synchronous high-grade malignant peripheral nerve sheath tumour (MPNST) and head and neck squamous cell carcinoma (HNSCC). This patient met the diagnostic criteria^4^ for LFS, but was wild-type for *TP53*. Additional sequencing and functional characterisation were performed to investigate other susceptibility genes potentially contributing to the cancers seen in this patient.

## Results

### Case report

**Figure 1:**
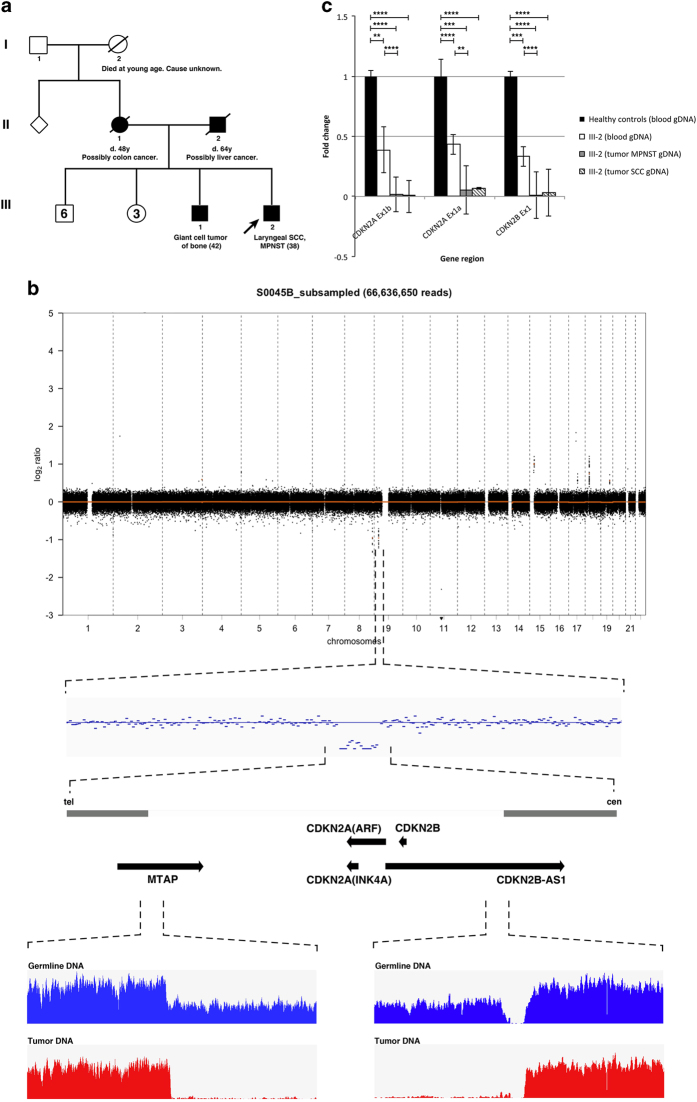
(**a**) Family pedigree of the LFL patient. Open and filled symbols represent unaffected and affected individuals respectively, with diagnosis and age of onset indicated under the symbols. Diagonal lines represent deceased individuals. Proband is marked by the arrow. (**b**) Copy-number analysis of sequenced patient germline DNA revealed focal deletion of the chromosome 9 encompassing the entire *CDKN2A–CDKN2B* locus. Sequential zooming in of the deleted locus is visualised from top (global copy-number plot) to bottom (sequencing coverage plot at the telomeric and centromeric breakpoints of locus). Genes affected by the deletion are represented by arrows. The sequencing coverage illustrated hemizygous and homozygous loss of the region in germline (blue) and MPNST tumour (red) DNA, respectively. cen, centromeric; tel, telomeric. (**c**) Real-time qPCR validated the *CDKN2A–CDKN2B* locus deletion. Coding regions for p14^ARF^, p16^INK4A^ and p15^INK4B^ were represented by *CDKN2A* exon 1b, 1a and *CDKN2B* exon1, respectively. The reduced gene dosage ratio in patient germline and tumours (MPNST, laryngeal SCC) DNA compared with a pool of three healthy controls reflected the hemizgygous and homozygous loss of this locus. Each data point is a mean of quintuplicates with s.e. presented as error bars. *P* value was computed using Student’s *t*-test. **P<0.01, ***P<0.001, ****P<0.0001.

Patient is a 39-year-old male initially diagnosed with moderately differentiated laryngeal squamous cell carcinoma (SCC) with subglottic extension staged as T2N2bM0. Subsequent positron emission tomography (PET) scan revealed a soft-tissue mass adjacent to the right humeral head and another large mass in the left iliopsoas extending to the left inguinal and femoral region, which was diagnosed as grade III MPNST. Apart from tobacco usage, the patient had no remarkable personal or medical record suggestive of high-risk exposure to laryngeal SCC. As his clinical presentation, together with known family history, met the Chompret criteria^4^ for LFS (Figure 1a), clinical genetic testing was performed for *TP53*. No pathogenic sequence or copy-number changes were found. However, in this test, *TP53* was part of a larger gene panel, which revealed a hemizygous deletion of all three p16^INK4A^ exons of *CDKN2A*. Given the absence of a personal or family history of melanoma, which might be expected in individuals with *CDKN2A* (specifically p16^INK4A^) mutations, we performed germline whole-genome sequencing (WGS) to further elucidate the basis for this patient’s disease.

### Whole-genome sequencing

Copy-number analysis of the WGS data revealed a constitutional focal deletion on chromosome 9 of ~270 kb, resulting in a hemizygous loss of the entire *CDKN2A–CDKN2B* locus and partially truncating the flanking *MTAP* and *CDKN2B-AS* genes (Figure 1b). This region is homozygously deleted in the tumour, as seen by the near-loss of sequencing coverage in the MPNST tumour DNA (Figure 1b). Quantitative PCR (qPCR) validated the deletions in the patient’s germline and tumour DNA, demonstrating a gene dosage ratio of 0.5 and 0.0, respectively, compared with healthy controls (Figure 1c). In addition, analysis of WGS data and PCR detection on the laryngeal SCC tumour (using PGMY-GP consensus primers,^5^ data not shown) did not indicate ongoing human papillomavirus infection.

### Molecular and functional analyses

**Figure 2:**
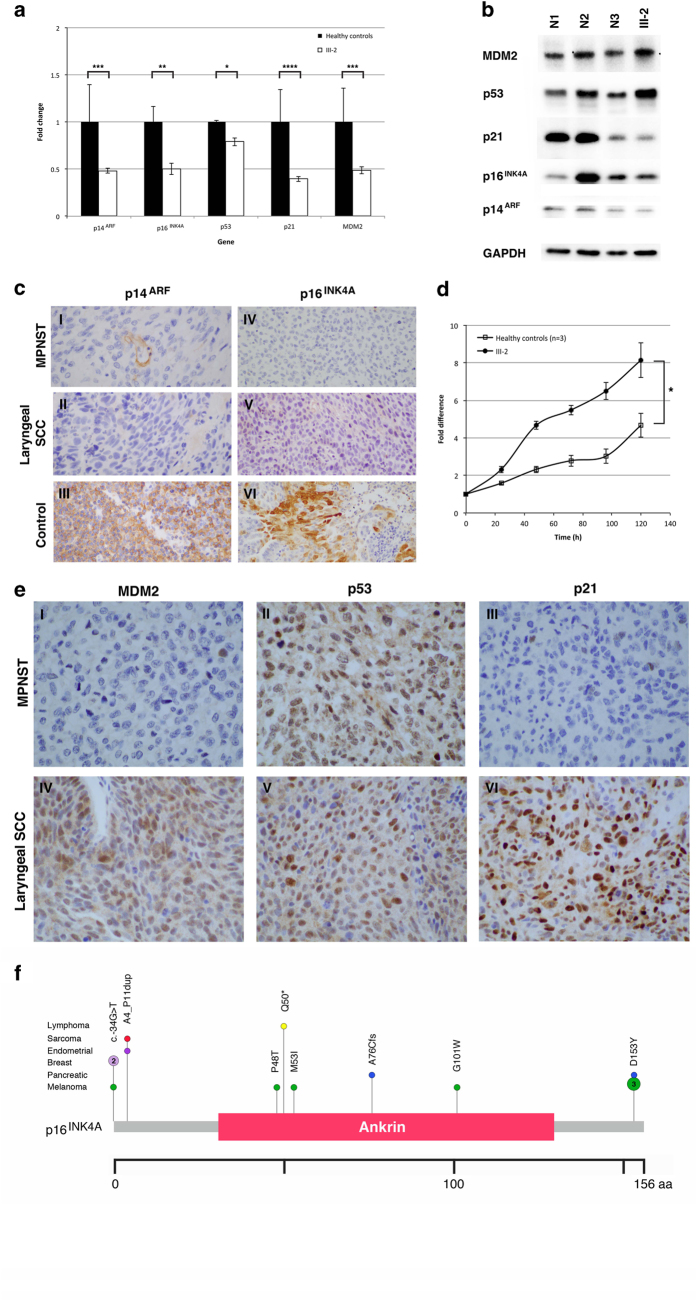
(**a**) Lower basal messenger RNA (mRNA) expression of *CDKN2A* and p53 pathway genes in LCL of the LFL patient (III-2) compared with a pool of three healthy controls. Fold change in mRNA was normalised against endogenous GAPDH and then compared against the mean of healthy controls. Each data point is a mean of triplicates with s.e. presented as error bars. *P* value was computed using Student’s *t*-test. *P<0.05, **P<0.01, ***P<0.001, ****P<0.0001. (**b**) Basal protein expression of *p14*^*ARF*^, *p16*^*INK4A*^, p53, MDM2 and p21 detected in whole-cell lysates derived from LCLs of the LFL patient compared with three healthy controls. GAPDH was used as loading control. N1, N2 and N3 represent healthy controls; III-2 represents LFL patient with *CDKN2A–CDKN2B* deletion. (**c**) Tumour sections of the LFL patient’s MPNST and laryngeal SCC were negatively stained for p14^ARF^ and p16^INK4A^, implying loss of the protein expression in these tumours. (I–III) Immunohistochemical (IHC) for p14^ARF^; (IV–V) IHC for p16^INK4A^. III and VI are tonsil and breast specimens, respectively, used as reference for positive control of the antibody staining. (**d**) Cell viability assay of the LFL patient compared against a pool of three healthy controls demonstrated significantly higher rate of cell proliferation. Assay was performed in quintuplicates. For healthy controls, LCLs from three healthy volunteers were used and the averaged value from these three lines were represented. Error bars: s.e. *P* value was computed using Student’s *t*-test. *P<0.05. (**e**) Differential MDM2 and p21 expression profiles in MPNST and laryngeal SCC tumours. These two proteins were undetectable in MPNST but expressed in laryngeal SCC tumour, suggesting distinct mechanisms potentially involved in tumorigenesis of the two tumours. However, p53 expression was observed in both tumours. (I–III) MPNST; (IV–VI) laryngeal SCC. (**f**) The tumour spectrum of additionally reviewed 13 patient cases with *CDKN2A* pathogenic/likely pathogenic variants presented in a lollipop schematic. The frequency of each phenotype is 1, unless indicated within the lollipop.

Hemizygous loss of *CDKN2A* could affect dosage of two tumour suppressor proteins encoded by the gene, *p14*^*ARF*^ and *p16*^*INK4A*^. To investigate, we compared the endogenous messenger RNA and protein expression levels in lymphoblastoid cell lines (LCLs) derived from the patient versus healthy controls. Reverse transcription qPCR analysis confirmed that basal gene expression of *p14*^*ARF*^ and *p16*^*INK4A*^ was 50% lower than in healthy controls (Figure 2a, *P*<0.001 and *P*<0.01, respectively). Immunoblot analysis of these two proteins in the patient LCLs did not show such a significant decrease; however, it should be noted that the p14^ARF^ and p16^INK4A^ levels in the LCLs of healthy controls were variable (Figure 2b). Nevertheless, immunohistochemical analysis of both MPNST and laryngeal SCC tumours were clearly null for p14^ARF^ and p16^INK4A^ (Figure 2c), consistent with the genomic loss observed by WGS (Figure 1b).

A germline hemizygous loss of *CDKN2A* associated with lower endogenous p14^ARF^ suggests possible p14^ARF^ haploinsufficiency, which may affect p53-dependent cell cycle control even in the absence of a *TP53* mutation in the patient.^6^ The observed significantly higher rate of cell proliferation in the patient LCL supports the possibility of deregulated cell cycle control (Figure 2d). To determine the potential consequences of reduced p14^ARF^ expression on the p53-dependent pathway, we investigated the expression levels of p53, MDM2 and one of the p53 downstream targets, p21. Whereas the messenger RNA and corresponding protein expression levels of MDM2 and p53 were inconsistent, p21 expression in the patient was significantly reduced at both messenger RNA and protein levels (Figures 2a and b). Interestingly, while all three proteins were expressed in the laryngeal SCC tumour, MDM2 and p21 were not detectable in the MPNST (Figure 2e).

## Discussion

To our knowledge, this is the first report of a hemizygous *CDKN2A–CDKN2B* germline deletion in an LFS/LFL family, where the proband presented with MPNST–HNSCC without a personal or family history of melanoma. The *CDKN2A–CDKN2B* locus encodes three tumour suppressor proteins—p14^ARF^, p16^INK4A^ and p15^INK4B^—all involved in cell cycle via p53 and retinoblastoma pathways.^7^ p16^INK4A^ and p15^INK4B^ inhibit cyclin-dependent kinase (CDK)-mediated phosphorylation of retinoblastoma, thereby promoting G1 cell cycle arrest.^7^ On the other hand, upon oncogenic stress, p14^ARF^ is induced and inhibits E3 ligase activity of MDM2 towards p53, thus leading to the stabilisation of p53 and activation of p53-mediated cell cycle arrest. As p14^ARF^ acts upstream of p53, it is conceivable that the germline hemizygous deletion of *CDKN2A* in our patient would phenocopy a hemizygous loss of *TP53* in the impaired capacity for arresting cell proliferation. Indeed, it has been alluded that p14^ARF^ haploinsufficiency may predispose to tumour formation.^8^ It has also been demonstrated that p14^ARF^-null and p14^ARF^-hemizygous mice are prone to earlier tumour development, especially of the sarcoma, lymphoma, carcinoma and neural system tumours spectrum,^9–11^ but to our knowledge, this patient is the first human model that includes p14^ARF^ deficiency. The consistent observation of significantly reduced gene and protein expression of the p53 downstream target p21 in our patient LCL suggests the possible deregulation of p21 (Figures 2a and b), supporting this hypothesis. We note the variability in protein expression among the LCLs of healthy controls, probably due to the variable effects of Epstein–Barr virus in the immortalisation of cell lines^12^ and are thus interpreted with caution. However, the loss of p21 expression in the MPNST revealed by immunohistochemical analysis (Figure 2e) further supports our hypothesis.

The complete genomic loss of the *CDKN2A* locus together with ablation of p14^ARF^ and p16^INK4A^ expression in both tumours (Figures 1b and 2c) is consistent with the observations in p14^ARF^-hemizygous mice, whereby loss of the residual wild-type *ARF* was observed to accompany tumour development.^9^ Interestingly, p21 and MDM2 were also undetectable in the MPNST with only p53 staining positive in the tumour, unlike the strong presence of all the three proteins in his laryngeal SCC. This differential protein expression between the patient’s MPNST and laryngeal SCC tumours suggests the driving mechanisms in tumorigenesis of these tumours are potentially distinct. Presence of all three p53 pathway-associated proteins in the laryngeal SCC tumour suggests that tumorigenesis was probably less dependent on the p53 pathway and could potentially be more driven by deregulation in the retinoblastoma pathway associated with loss of p16^INK4A^.^13^ In contrast, in the MPNST, expression of p21 appears to be abrogated even in the presence of p53 (Figure 2e), suggesting deregulation of p21 potentially could be indirectly mediated by p14^ARF^ via other transcriptional factors in a p53-independent manner^14,15^ and phenocopy LFS. Although it is known that p14^ARF^ predominantly inhibits MDM2 thereby stabilizing p53 and promoting its activation,^16^ emerging body of evidences has shown that p14^ARF^ can interact with a host of proteins that may mediate the tumour suppressor activities of p14^ARF^^17^ independent of p53.

Germline mutations in *CDKN2A* are associated with familial melanoma and pancreatic cancer, and infrequently with neurofibroma,^18^ HNSCC^19^ and neural system tumours (astrocytoma and glioma).^20^ To date, *CDKN2A* deletions have been reported in only a handful of kindreds displaying clustering of specific tumour spectrum, all of which feature melanoma: cutaneous malignant melanoma-neural system tumour,^20–22^ melanoma-HNSCC,^19^ melanoma-neurofibroma^18^ and malignant melanoma.^23,24^ We reviewed the clinical records for a series of cancer patients who were found to be positive through gene panel testing for germline *CDKN2A* mutations to better understand the phenotypic and mutation spectrum. In this series, there were a total of 29 cancers reported among the 14 patients from 12 unique families with germline *CDKN2A* mutations. The most common cancer was melanoma occurring in seven patients followed by sarcoma, pancreatic and breast cancers each occurring in two patients (Figure 2f, Supplementary Table 1). The overall average age at first cancer diagnosis was 34 years (age range 9–60 years) and the average age at diagnosis of first melanoma was 27 years. Three patients had a history of multiple melanomas and one developed pancreatic cancer 25 years after a melanoma diagnosis. One homozygous individual presented with Hodgkin’s lymphoma at age 12 years and had family history of early-onset lymphoma, jaw tumours, pancreatic, breast and lung cancers. A family history that would suggest hereditary cancer risk was reported in 13 out of 14 patients. Importantly, 2 out of 14 patients met the Chompret criteria and 11 out of 14 reported a family history of cancers characteristic of LFS/LFL spectrum. A review by Baker *et al.* highlighted a rare contiguous deletion in chromosome 9p21.3 obliterating ~25 genes including *CDKN2A*, *CDKN2B* and *MTAP* in a melanoma-prone family with additional tumour spectrum (neural system tumours, leukaemia, chondrosarcoma, pontomedullary PNET and cervical cancer),^25^ supporting our findings that genetic alterations involving *CDKN2A* could potentially underlie hereditary predisposition to cancers beyond melanoma. In our patient with hemizygous germline deletion of *CDKN2A*, it is prudent to extend melanoma screening given the increased risk of melanoma in families with *CDKN2A* alterations.

Recent reports revealed that loss of *MTAP*, a gene flanking and frequently co-deleted with *CDKN2A*, can render cancer cells sensitive to PRMT5 inhibition, thus opening up opportunities to exploit this vulnerability for treating tumours with *MTAP–CDKN2A* co-deletion.^26^ For affected individuals with large deletions involving *CDKN2A* and *MTAP*, such as our patient and the family described by Baker *et al.*,^25^ the possibility of targeting this PRMT5 dependence raises potential for therapeutic strategies.

Currently, not all clinical testing panels include the entire *CDKN2A* locus (encompassing both p14^ARF^ and p16^INK4A^ genes). Our study suggests the importance of interrogating the full extent of this locus as alterations to p14^ARF^ may account for a small subset of germline *TP53* wild-type LFS/LFL cases and should be included in clinical testing gene panels.

In summary, our study highlights the contribution of *CDKN2A* germline deletion to cancer predisposition in LFS/LFL patients and expands the non-melanoma phenotypic spectrum of cancers associated with germline *CDKN2A* mutations. Clinicians should consider genetic testing, including the entire *CDKN2A* locus, in their patients with LFS/ LFL or those with personal history of sarcoma, and family history of cancers beyond the traditional spectrum of *CDKN2A*. To the best of our knowledge, this is also the first report of a human p14^ARF^ deficiency model, providing insights into the potential role of p14^ARF^ and deregulation of the p53 pathway in sarcoma tumour development.

## Materials and methods

This study was approved by the SingHealth Centralised Institutional Review Board (IRB 2011/826/B). Signed informed consent was given by the patient.

### Genetic analysis

Clinical genetic testing was performed at Invitae as previously described.^27^ WGS of patient genomic DNA purified from peripheral blood and MPNST tissue was performed on an Illumina Hiseq2000 (Illumina Inc., San Diego, CA, USA) to a mean coverage of 72×, data analysed by in-house pipeline and deposited in the European Nucleotide Archive (accession no. PRJEB13761). The genomic deletion was validated by qPCR. For detailed methods, refer to Supplementary Information.

### Functional studies

LCLs established by Epstein–Barr virus immortalisation of peripheral blood mononuclear cells of patient and healthy volunteers were used for cell proliferation and expression studies. Cell proliferation was assessed by viability assay using the CellTiter-Glo Luminescent Cell Viability Assay kit (Promega, Madison, WI, USA), whereas gene expression was quantitated using messenger RNA extracted from LCLs by reverse transcription qPCR. Protein expression was assessed by immunoblotting of whole-cell lysates from LCLs. Detailed methods including reverse transcription qPCR primers and immunoblotting antibodies are described in Supplementary Information. Immunohistochemical analysis was performed on 2-μm-thick slides sectioned from patient tumours and scored by a pathologist.

### Statistical analysis

*P* values were computed using two-tailed Student’s *t*-test and were not adjusted for multiple testing.
