# Supplementary material for: Germline hemizygous deletion of CDKN2A–CDKN2B locus in a patient presenting with Li–Fraumeni syndrome
Source: NPJ Genom Med. 2016 Jun 1;1:16015–. doi: 10.1038/npjgenmed.2016.15 (PMC5685299; doi:10.1038/npjgenmed.2016.15)
Supplement: Supplementary Information [file npjgenmed201615-s1.doc]

**Supplementary Information**

Chan *et. al* Germline Hemizygous Deletion of *CDKN2A-CDKN2B* Locus in a Patient Presenting with Li-Fraumeni Syndrome

Contents

Supplemental Methods [1](#__RefHeading___Toc322375880)

Clinical genetic testing [1](#__RefHeading___Toc322375881)

Nucleic acid extraction [1](#__RefHeading___Toc322375882)

Whole genome sequencing [2](#__RefHeading___Toc322375883)

Cell culture [3](#__RefHeading___Toc322375884)

Cell viability assay [3](#__RefHeading___Toc322375885)

Quantitative (reverse transcription) polymerase chain reaction ((RT-)qPCR) [4](#__RefHeading___Toc322375886)

Primer sequences for qPCR & RT-qPCR [4](#__RefHeading___Toc322375887)

Immunoblot analysis [5](#__RefHeading___Toc322375888)

Analysis of additional patient cases [6](#__RefHeading___Toc322375889)

Supplementary Table 1 [7](#__RefHeading___Toc322375890)

Supplementary References [8](#__RefHeading___Toc322375891)

# Supplemental Methods

## Clinical genetic testing

Clinical genetic testing was performed at Invitae as previously described^1^. The genes evaluated were:

*APC* (NM_000038.5), *ATM* (NM_000051.3), *BMPR1A* (NM_004329.2), *BRCA1* (NM_007294.3), *BRCA2* (NM_000059.3), *BRIP1* (NM_032043.2), *CDH1* (NM_004360.3), *CDK4* (NM_000075.3), *CDKN2A* (NM_000077.4), *CHEK2* (NM_007194.3 (c.1100delC only)), *EPCAM* (NM_002354.2), *FANCA* (NM_000135.2), *FANCB* (NM_001018113.1), *FANCC* (NM_000136.2), *FANCE* (NM_021922.2), *FANCF* (NM_022725.3), *FANCG* (NM_004629.1), *FANCI* (NM_001113378.1), *FANCL* (NM_018062.3), *MEN1* (NM_130799.2), *MET* (NM_001127500.1), *MLH1* (NM_000249.3), *MSH2* (NM_000251.2), *MSH6* (NM_000179.2), *MUTYH* (NM_001128425.1), *NBN* (NM_002485.4), *NF1* (NM_000267.3), *PALB2* (NM_024675.3), *PALLD* (NM_001166110.1), *PMS2* (NM_000535.5), *PTCH1* (NM_000264.3), *PTEN* (NM_000314.4), *RAD51C* (NM_058216.1), *RET* (NM_020975.4), *SLX4* (NM_032444.2), *SMAD4* (NM_005359.5), *SPRED1* (NM_152594.2), *STK11* (NM_000455.4), *TP53* (NM_000546.5), *VHL* (NM_000551.3)

## Nucleic acid extraction

Patient genomic DNA was purified from peripheral blood using Blood & Cell Culture kit (Qiagen, Hilden, Germany), fresh frozen MPNST tissue using QIAamp DNA mini kit (Qiagen, Hilden, Germany) and formalin-fixed paraffin embedded (FFPE) laryngeal SCC tissue using QIAamp DNA FFPE Tissue kit (Qiagen, Hilden, Germany), according to manufacturer’s protocol. Purified genomic DNA was subsequently used for whole genome sequencing and quantitatve PCR (qPCR).

mRNA from lymphoblastoid cell lines (LCL) were extracted using Rneasy Mini kit (Qiagen, Hilden, Germany) and DNA synthesized using High Capacity mRNA-to-cDNA kit (Applied Biosystems, Carlsbad, CA, USA) for quantitative reverse transcription PCR (RT-qPCR).

## Whole genome sequencing

Whole genome sequencing (WGS) was performed by Macrogen (Korea) on Illumina Hiseq2000 (Illumina Inc., San Diego, CA, USA) using paired-end 151-base pair reads. Alignment of sequencing reads to the human reference genome (hs37d5) and variant calling was performed using the SpeedSeq pipeline. Briefly, reads were aligned to the reference genome using the Burrows-Wheeler Aligner (BWA) version 0.7.10. Marking of duplicate reads and read sorting were performed using SAMBLASTER version 0.1.22 and Sambamba version 0.5.4 respectively. Freebayes version 0.9.21 was then used to call both germline variants (using the blood sample) and somatic variants (using both tumor and blood samples) (Supplemental Table 1). The default settings provided by the SpeedSeq pipeline were used. Mean sequencing depth was 72-73X, with 91-92% of the genome covered by at least 20 reads. Variants were filtered by read depth (≥ 20X). For germline variants, a minimum variant allele frequency (VAF) of 20% was required, whereas for somatic variants, a minimum VAF of 5% was required. Identification of copy number variations (CNVs) in the samples was performed using the QDNAseq tool. To improve computational efficiency, copy number calling was performed using 5% of total reads in each sample that were randomly subsampled. For prioritization of candidate germline variants, the following approach was used. First, the variant VCF file was annotated using the wAnnovar web application (http://wannovar.usc.edu/). Variants were filtered to retain only non-synonymous exonic variants as well as splice site variants. To remove common polymorphisms, we excluded variants present in more than 1% of the population as defined by the ExAC and 1000 genomes databases^2,3^.

## Cell culture

Lymphoblastoid cell lines (LCLs) were established by EBV-immortalization of peripheral blood mononuclear cells (PBMC) derived from 8 mililiter (ml) whole blood of patient and healthy volunteers. LCLs were maintained in RPMI-1640 growth medium (Gibco, Gaithersburg, MD, USA) supplemented with 20% fetal bovine serum (Hyclone, Logan, UT, USA), 1X antimycotic-antibiotics (Gibco) and incubated at 37°C with 5% CO_2_. Each line was passaged once every 3-5 days for no more than 15-20 passage numbers.

## Cell viability assay

Cell viability was assessed by ATP assay using CellTiter-Glo Luminescent Cell Viability Assay kit (Promega, Madison, WI, USA). LCLs were seeded in 96-well flat-bottom assay plates (Costar, Kennebunk, ME, USA) in 100 microliter (μl) growth medium at a density of 1 x 10^3^ cells per well and incubated at 37°C. CellTiter-Glo Reagent was added in 50 μl aliquots per well at each assay time point, briefly incubated at room temperature and luminescence recorded on Tecan Infinite200 PRO microplate reader (Tecan, Männedorf, Switzerland). Quintuplicate was performed for each line. Fold change in cell viability was normalized against measurement at time 0 hour (h).

## Quantitative (reverse transcription) polymerase chain reaction ((RT-)qPCR)

qPCR and RT-qPCR were performed in 10 μl aliquots using 40-50 nanogram (ng) genomic DNA or complementary DNA (cDNA), respectively, with Ssofast Evagreen Supermix (Bio-rad, Hercules, CA, USA) on CFX96 Real-Time PCR Detection System (Bio-rad). Triplicates were prepared for each sample. Cycling conditions used: 30 seconds (s) at 98°C for enzyme activation, followed by 40 cycles of 5 s at 98°C and 5 s at 60°C for denaturation and extension, finally completed with melt curve analysis at 65°C-95°C. Cycle threshold (C_t_) values were normalized to GAPDH endogenous control and the fold change in gene expression calculated using the ΔΔC_t_ method by normalizing against healthy controls.

## Primer sequences for qPCR & RT-qPCR

For detection of gene dosage in genomic DNA:

| **Gene** | **Primer sequence (5’ – 3’)** | **Source** |
| --- | --- | --- |
| *CDKN2A* (Exon 1b) | Fw: CTC GTG CTG ATG CTA CTG AGG A  Rv: TTA GAA GCT CTG TTC GCC TCA G | Gonin-Laurent et. al |
| *CDKN2A* (Exon 1a) | Fw: CTC CAG AGG ATT TGA GGG ACA G  Rv: CTC TTT CTT CCT CCG GTG CTG | Gonin-Laurent et. al^4^ |
| *CDKN2B* (Exon 1) | Fw: CGT TAA GTT TAC GGC CAA CG  Rv: CGC ACC TTC TCC ACT AGT CC | Lindberg et. al^5^ |
| GAPDH | Fw: CAT CCC TTC TCC CCA CAC AC  Rv: CTA GTC CCA GGG CTT TGA TTT G | Gonin-Laurent et. al^4^ |

For assessment of mRNA expression:

| **Gene** | **Primer sequence (5’ – 3’)** | **Source** |
| --- | --- | --- |
| p14^ARF^ | Fw: CTC GTG CTG ATG CTA CTG AGG  Rv: CCC ATC ATC ATG ACC TGG TCT T | Gonin-Laurent et. al^4^ |
| p16^INK4A^ | Fw: CCA ACG CAC CGA ATA GTT ACG  Rv: CCA TCA TCA TGA ACC TGG ATC G | Gonin-Laurent et. al^4^ |
| p53 | Fw: TCA ACA AGA TGT TTT GCC AAC TG  Rv: ATG TGC TGT GAC TGC TTG TAG ATG | Teoh et. al^6^ |
| p21 | Fw: GCA GAC CAG CAT GAC AGA TTT  Rv: GGA TTA GGG CTT CCT CTT GGA | Saramaki et. al^7^ |
| MDM2 | Fw: ACC TCA CAG ATT CCA GCT T  Rv: GCA ATG GCT TTG GTC TAA CCT | Gonin-Laurent et. al^4^ |
| GAPDH | Fw: GAA GGT CGG AGT CAA CGG ATT  Rv: TGA CGG TGC CAT GGA ATT TG | Gonin-Laurent et. al^4^ |

## Immunoblot analysis

Whole cell lysates were extracted from LCLs using radioimmunoprecipitation assay (RIPA) lysis buffer [100 nM sodium fluoride (NaF), 1 nM sodium orthovanadate (Na_3_VO_4_), 10mM phenylmethanesufonyl fluoride (PMSF) in basic RIPA lysis buffer] supplemented with protease inhibitor cocktail (Roche, Basel, Switzerland). Total protein of 40 microgram (μg) was separated by SDS-PAGE, transferred to PVDF membrane and probed with the antibodies tabled below. Blots were developed using Amersham ECL Primer Western Blotting Detection Reagent (GE Healthcare) on ChemiDoc MP System (Bio-rad).

| **Antibody** | **Host species** | **Source** | **Dilution** |
| --- | --- | --- | --- |
| p14^ARF^ | Rabbit | Kind gift from Koji Itahana | 1:500 |
| p16^INK4A^ (G175-1239) | Mouse | BD Pharmingen | 1:500 |
| p53 (DO-1) | Mouse | Santa Cruz Biotechnology | 1:1,000 |
| p21 (SX118) | Mouse | Kind gift from Stephan Gasser | 1:500 |
| MDM2 (2A9) | Mouse | Kind gift from TP53 lab | 1:500 |
| β-actin (AC-15) | Mouse | Sigma-Aldrich | 1:10,000 |
| Anti-mouse IgG, HRP-linked | Sheep | GE Healthcare | 1:2,000 |
| Anti-rabbit IgG, HRP-linked | Donkey | GE Healthcare | 1:4,000 |

## Analysis of additional patient cases

Patient cases were selected for analysis, from individuals referred to Invitae for multi-gene panel genetic testing with a personal and/or family history of cancer, if they had a Pathogenic or Likely Pathogenic variant in the p16^INK4A^ reading frame of *CDKN2A*. Variants were classified using a point-based system that closely adhered to the American College of Medical Genetics and Genomics (ACMG) guidelines. De-identified personal and family histories provided by ordering clinicians were examined and tabulated in Supplementary Table 1.

# Supplementary Table 1

| ***Patient no.*** | ***Variant*** | | ***Zygosity*** | ***Proband cancer history*** | ***Age of diagnosis (years)*** |
| --- | --- | --- | --- | --- | --- |
|  | ***Genomic change*** | ***Protein change*** |  |  |  |
| 1 | c.-34G>T | Non-coding | Het | Breast | 35 |
| 2 | c.-34G>T | Non-coding | Het | Breast | 56 |
| 3 | c.-34G>T | Non-coding | Het | Melanoma (x3)^ | 19, 22, 24 |
| 4 | c.9_32dup | p.A4_P11dup | Het | Endometrial | 60 |
| 5 | c.9_32dup | p.A4_P11dup | Het | Osteosarcoma | 9 |
| 6 | c.142C>A | p.P48T | Het | Melanoma | 22 |
| 7 | c.148C>T | p.Q50* | Hom | Hodgkin’s lymphoma | 12 |
| 8 | c.159G>C | p.Met53Ile | Het | Melanoma (x10)^ | 22-70 |
| 9 | c.225_243del | p.A76Cfs*64 | Het | Pancreatic | 56 |
| 10 | c.301G>T | p.G101W | Het | Melanoma (x4)^ | 16, 21, 25, 35 |
| 11 | c.457G>T | p.D153Y | Het | Melanoma, Pancreatic | 50, 75 |
| 12 | c.457G>T | p.D153Y | Het | Melanoma | 28 |
| 13 | c.457G>T | p.D153Y | Het | Melanoma | 32 |
| 14* | Whole gene deletion |  | Het | Sarcoma (x2)^, Laryngeal SCC | 38 |

Supplementary Table1: List of 14 patient cases with *CDKN2A* pathogenic/likely pathogenic variants and the associated tumor spectrum. Het: heterozygous, Hom: homozygous, ^: parentheses indicates the number of separate diagnoses in the patient, *: patient is III-2 in this case report. Patients 1 and 5 met the Chompret criteria for Li-Fraumeni Syndrome. SCC: squamous cell carcinoma.

# Supplementary References

1. Lincoln SE, Kobayashi Y, Anderson MJ, et al. A Systematic Comparison of Traditional and Multigene Panel Testing for Hereditary Breast and Ovarian Cancer Genes in More Than 1000 Patients. J Mol Diagn JMD 2015;17(5):533–44.

2. Consortium T 1000 GP. An integrated map of genetic variation from 1,092 human genomes. Nature 2012;491(7422):56–65.

3. Exome Aggregation Consortium (ExAC), Cambridge, MA [Internet]. Available from: http://exac.broadinstitute.org

4. Gonin-Laurent N, Hadj-Hamou NS, Vogt N, et al. RB1 and TP53 pathways in radiation-induced sarcomas. Oncogene 2007;26(41):6106–12.

5. Lindberg D, Akerström G, Westin G. Evaluation of CDKN2C/p18, CDKN1B/p27 and CDKN2B/p15 mRNA expression, and CpG methylation status in sporadic and MEN1-associated pancreatic endocrine tumours. Clin Endocrinol (Oxf) 2008;68(2):271–7.

6. Teoh PJ, Chung TH, Sebastian S, et al. p53 haploinsufficiency and functional abnormalities in multiple myeloma. Leukemia 2014;28(10):2066–74.

7. Saramäki A, Banwell CM, Campbell MJ, Carlberg C. Regulation of the human p21(waf1/cip1) gene promoter via multiple binding sites for p53 and the vitamin D3 receptor. Nucleic Acids Res 2006;34(2):543–54.
